# Everybody's Page

**Published:** 1888-09-22

**Authors:** 


### Everybody's Page.

In the prison of Leopoldstadt, at Vienna, which was very badly ventilated, there died between the years 1834 and 1847, 378 prisoners out of 4,280, or 86 per 1,000; and of these no less than 220, or 51·4 per 1,000 died from consumption.

The night air in towns is often the purest. In some country places, as in the fens, there may be malaria, which may cause ague; but in most towns there is no danger in breathing night air from without; the real danger is from the vitiated night air within doors.

Intellectually, the dyspeptic is oftener than most men below the mark. A man cannot do the best mental work he is capable of when in actual pain or in low spirits. A great historical illustration of this thesis is the loss of the battle of Leipsic by Napoleon, because, so it is said, he was suffering from an attack of dyspepsia brought on by a hurried meal from a shoulder of mutton and onions.

The history of the epidemics of the middle ages, as given by Hecker, is one of the most fearful and murderous that can be imagined, the numbers carried off by them far exceeding those slain in the most bloody war ever carried on. In Italy three-fourths of the population died of the "Black Death" spoken of by Bocaccio; in Paris, 50,000; in London, at least 100,000; and in Norwich alone, 51,100.

Excursions to the seaside, and other such places to which healthseekers resort, are now within the means of the poorest. Is it not a fact, however, that cheap trips, so called, are but too often inverted in their consequences by perversity of the trippers? Bad beer, worse gin, tobacco fumes, and riot constantly antagonise the beneficial influences that ought to flow from these excursions.

An instrument called the osteotome has been invented as an improvement over the tedious and clumsy surgical methods of cutting through bones by means of saws and chisels. It is practically a circular saw revolving at very high speed by an electric motor. An ingeniously-devised shield, which passes around the bone to be operated upon, protects the surrounding flesh from injury.

Among trades that tend to shorten life is that of knife grinding, as practised in the great cutlery factories of Sheffield. A fork grinder once said to Dr. J. S. Hall, of Sheffield, "I shall be thirty-six next month, and you know that is getting an old man at our trade." Another, a young man of about twenty-six, said, "He reckoned in about two more years, at his trade, he might begin to think of dropping off the perch;" adding, "You know a knife grinder is an old cock at thirty."

In Manchester, in the year 1871, 9,739 children were born alive. In the same year 2,075 infants died. The proportion of deaths of infants to births was therefore 213 per 1,000. The waste of infant life in Manchester, as compared with the rates for the whole of England and Wales, amounted to 535 children under one year of age. If the Manchester mortality is compared with that of healthy districts, the waste amounted to 10·70 lives.

Mental calm, pure air, and suitable food taken at proper intervals, with moderate but systematic muscular exercise out of doors, form essential factors in the maintenance of a sound mind in a sound body, the general prevalence of which character of mind and body would realise the desiderated state of health in the community. These factors constitute the special agencies having a definite relation with the human organism, which, in their absence, must certainly deteriorate and prematurely perish.

When exercise is taken the circulation is quickened, and the skin becomes warm and perspiring. Then, when it is over, the skin soon loses heat and becomes very susceptible of cold, and a chill is easily caught. It is necessary, therefore, during exercise or when at work, to wear light clothing, and when it ceases something of a woollen material in addition.

In the period of growth, mineral substances, such as the phosphates of lime and magnesia, are needed especially for building up the bony framework of the body. Oatmeal, wheat, and maize contain these phosphates; but fine wheat flour has a great part removed, so that what is called the best is not the best for the growth of the body. The browner flour is more nutritious, and if it were only ground fine enough, the best and most nutritious would be the whole grain ground down together.

The *Courier de l'Europe* records a shower of blackish mud which fell at Naples on March 17th. Professor Palmieri shows that the phenomena is very simple in its origin. Violent winds in Africa raise up columns of dust and sand, which are swept over the south of Europe. It readily happens that rain, falling through these clouds, is rendered turbid. If the dust is black there is a shower of mud. But if it is red, ferruginous matter, the rain is red, and we have the still more alarming phenomenon of a "shower of blood."

A Russian physician, named Portugaloff, declares that strychnine is an infallible cure for drunkenness, administered in subcutaneous injections. He asserts that the experience of physicians has shown the cure to be as rapid as it is certain. The effect of the strychnine solution is to change the craving for drink into positive aversion, and this change is effected in a day. After a treatment of eight or ten days the patient may be discharged. The strychnine is administered by dissolving one grain in two hundred drops of water, and injecting five drops of the solution every twenty-four hours.

Small-pox has carried off its hundreds, and tens of hundreds of thousands. Before the discovery of that beneficent resource, vaccination, we are told by Dr. Barlow that its share in all the deaths was one in every ten, and that of children under ten years of age one third were its victims. As Lord Macaulay has said, "The small-pox was always present, filling the churchyards with corpses, tormenting with constant fears all whom it had not yet stricken, leaving on those whose lives it spared the hideous traces of its power, turning the babe into a changeling at which the mother shuddered, and making the eyes and cheeks of the betrothed maiden objects of horror to the lover."

The British barque *Douglass* has arrived at Philadelphia from Monte Video, after an experience which makes the imaginary or plagiarised horrors of Rider Haggard seem tame. A cargo of bones was shipped, and the bones were not properly cleaned. They bred loathsome bugs, which drove the crew nearly crazy with disgust. The ship was alive with these crawling torments. They crept into the eyes of waking men, into the ears and mouths of those who tried to sleep. Swept off, washed away, stifled with burned sulphur, they swarmed again in undiminished numbers. Even in port, after weeks of effort, barrels of the bugs were taken out of the hold and the decks were covered with them. The sailors say that, of all the terrors of the sea, this was the worst they had ever encountered, and their gaunt figures, drawn faces, and discoloured skin prove that their sufferings were not merely mental.

